# Topical Retinoids in Acne Vulgaris and Acne Scars—From Monotherapy to Combining Regimens

**DOI:** 10.3390/ph19040620

**Published:** 2026-04-15

**Authors:** Aleksandra Tobiasz, Alina Jankowska-Konsur, Danuta Nowicka

**Affiliations:** University Centre of General Dermatology and Oncodermatology, Faculty of Medicine, Wroclaw Medical University, 50-556 Wroclaw, Poland; alina.jankowska-konsur@umw.edu.pl (A.J.-K.); danuta.nowicka@umw.edu.pl (D.N.)

**Keywords:** retinoid, acne, scar, combination treatment

## Abstract

Topical retinoids are the cornerstone of the treatment of multiple dermatological conditions. Long established in acne therapy, they exert effects on keratinization, inflammation, fibroblast activity, and collagen remodeling, suggesting a potential role in both the prevention and treatment of acne scars. This narrative review summarizes current evidence on the use of topical retinoids in acne vulgaris and acne scarring, focusing on different retinoid molecules, formulation technologies, and combination strategies. A review of published clinical and experimental studies evaluating tretinoin, adapalene, tazarotene, and trifarotene was performed, including their use as monotherapy and in combination with other topical agents or procedural interventions. The available data indicate that topical retinoids have a well-established position in acne treatment, can improve the appearance of atrophic acne scars, reduce the progression of scarring, and support skin remodeling. Advances in formulation technologies have improved tolerability, while combination approaches with agents such as benzoyl peroxide, antibiotics or procedural techniques have shown additive or synergistic effects, particularly in more severe cases. Nevertheless, much of the evidence regarding novel formulations is derived from small or heterogeneous study populations. In conclusion, topical retinoids represent a relevant therapeutic option in acne vulgaris and acne scarring, from monotherapy in mild cases to components of multimodal treatment protocols in more severe disease. Further large-scale, comparative studies are needed to better define the optimal clinical use of advanced drug delivery systems for topical retinoids.

## 1. Introduction

Vitamin A and its derivatives have been known in medicine for a long time and have been studied thoroughly since the 20th century [1]. As vitamin A has a significant role in epithelial integrity, retinoids administered topically and orally are widely used in the treatment of a wide range of dermatological conditions [2]. The term retinoids encompasses both natural and synthetic substances that share functional and structural similarities with vitamin A. Retinol binds to intracellular retinol-binding proteins, which facilitate its transport within cells and its conversion to retinoic acid. Because retinoids are lipophilic and poorly soluble in body fluids, they require carrier proteins, such as retinol-binding protein in complex with transthyretin, to enable their circulation in the bloodstream [3]. Their mechanism of action is dependent on activating nuclear receptors, which are retinoic acid receptors (RARs) and retinoid X receptors (RXRs). As a next step, they regulate the transcription process and therefore influence the expression of certain genes. As a result of this process, retinoids create changes in protein synthesis and, in such a way, impact cell proliferation. Retinoids are classified into four generations sharing some similarities in structure and properties. These generations are as follows:

1st—Retinol, tretinoin, isotretinoin (mainly used as oral treatment, but also recommended as topical treatment [4]), and alitretinoin, which are natural retinoids.

2nd—Etretinate and acitretin, which are not used topically.

3rd—Adapalene, bexarotene, and tazarotene, which are more selective toward retinoid receptors.

4th—Containing only one agent so far–trifarotene—a highly specific agent towards RAR-gamma receptors [5,6].

Characteristics of each of the topical retinoids have been summarized in Table 1. 

The use of topical retinoids is versatile, with one of their most common applications being the treatment of acne vulgaris. Because acne scarring represents one of the most burdensome long-term complications of acne, topical retinoids have also established a role in this indication. Their beneficial effects in acne vulgaris are primarily mediated through the regulation of keratinization and anti-inflammatory activity, leading to a reduction in microcomedone formation and the limitation of conditions favorable for *Cutibacterium acnes* proliferation.

Additionally, because of their collagen-stimulating and skin remodeling potential, effectiveness in acne scarring is observed [13,14].

The field of topical drug delivery is undergoing continuous and dynamic development. The aim of such formulations is to achieve maximal results with minimal irritation. Novelties such as nanostructured lipid carriers (NLCs), microemulsions, microemulgels and many others unlock new therapeutic potential for retinoids [15,16]. Given the lack of comprehensive synthesis linking retinoid mechanisms and formulation advances, particularly in the field of combination therapies, we conducted this review with the focus on summarizing the therapeutic potential of topical retinoids in acne vulgaris and in acne scarring, considering their use as monotherapy as well as in different combinations.

## 2. Materials and Methods

To ensure proper coverage, literature searches were conducted in PubMed/MEDLINE and Google Scholar. The following keywords and their combinations were applied: retinoid, acne vulgaris, acne scar, monotherapy, combination therapy, adjunct therapy, retinol, tretinoin, isotretinoin, adapalene, tazarotene, and trifarotene. No time restrictions were applied, and only articles published in English were considered. Eligible studies included clinical trials, in vivo and in vitro experimental studies. Studies not related to acne or acne scarring, as well as non-relevant publications, were excluded. The search focused on novel, innovative approaches towards topical retinoid therapy, which is why, besides larger studies, early-stage, smaller studies were included as well. Such an approach enabled the identification of emerging and newly developing trends in topical retinoid therapy.

## 3. First Generation—Tretinoin

Tretinoin—the first topical retinoid to be approved in 1971 for the treatment of acne vulgaris. Since that moment, it has become a cornerstone in dermatology. Despite being more than 50 years old, the molecule continues to demonstrate substantial therapeutic potential, as evidenced by ongoing studies exploring novel formulations. Over the years, tretinoin formulations have evolved, starting with a 0.05% tretinoin solution, and gradually introducing formulations such as cream, gel, and lotion in varying concentrations between 0.01% and 0.1%. Such modifications aimed to obtain a formulation of maximal effectiveness and tolerability [1,17,18,19,20].

Tretinoin, being in fact all-trans retinoic acid (ATRA), has strong binding potential with all three types of RARs (alpha, beta, and gamma). As a result of such non-selective action, severe side effects can be observed; however, they are typically localized and dose-dependent. Typical side effects that accompany tretinoin therapy, and in fact can, in certain conditions, appear during therapy with all kinds of topical retinoids, are erythema, peeling, dryness, oedema, burning, and itching sensation. Such side effects are the most common [21,22,23].

An interesting observation has been made by Martinez et al., suggesting that tretinoin beneficially alters the skin microbiome, which is an additional therapeutic effect [24]. Of course, such a topic requires more studies, but as correlations between skin microbiome and different dermatological diseases have been researched in recent years, finding a link between retinoid use and skin microbiota is of great impact.

Tretinoin contains multiple double bonds and remains a relatively unstable molecule. The most recent innovation remains tretinoin lotion at a concentration of 0.05%. Such a formulation represents a novel type of emulsion in which polymerization technology has been employed. In such an emulsion, micronized tretinoin is entrapped in a polymeric matrix of honeycomb structure. As a result, it is more resistant to various factors affecting its stability. Additionally, the honeycomb structure enables gradual drug release. Its favorable effects were confirmed in numerous studies [25,26,27,28,29]. It has now been officially approved in the United States for use in patients aged 9 years and older [30].

Because adults with acne constitute a large patient population, Stein et al. [27] conducted a study focusing on females with moderate to severe acne across different age groups. The results demonstrated that the greatest therapeutic benefit from tretinoin lotion was observed in women aged 30 years and older. On the other hand, the study by Bhatia et al. [31] confirmed good combining potential of tretinoin lotion with makeup. Such studies are of great importance, keeping in mind that adult patients are a demanding group of patients, whose functioning can be affected by acne to a large extent [32,33].

Polymeric emulsion technology remains the major technological advancement; however, at the same time, other novel, promising formulation advancements are being described.

One such approach involves novel tretinoin-loaded nanostructured lipid carriers (NLCs), in which the emulsion is formulated using a high-pressure homogenization process. NLC using a mixture of solid and fluid lipids ensures better stability, absorption, and optimal drug release. In the study conducted by Samadi et al. [34], a novel 0.05% NLC-tretinoin formulation was compared to a 0.05% tretinoin-loaded cream, showing that both formulations significantly reduced lesions on both sides of the face, but the reduction in non-inflammatory lesions was significantly greater on the tretinoin-loaded NLC-treated side. Not only was the clinical effect greater with the tretinoin-loaded NLC, but patient satisfaction with the product was also higher. However, the study was conducted in a small patient cohort, highlighting the need for validation in larger populations.

On the other hand, Malavi et al. [35] proposed entrapping tretinoin in an emulgel formulation, hypothesizing that this approach could offer improved stability and reduced local adverse effects. The authors successfully developed a stable formulation with favorable tolerability; however, the experiments were conducted in an animal model, and further human studies are required.

Novel guidelines of the American Academy of Dermatology regarding treatment of acne vulgaris mention the use of topical retinoids at every stage of acne severity. These recommendations apply to adults, adolescents, and preadolescents aged 9 years and older. Moreover, fixed-dose combinations of topical retinoids with benzoyl peroxide or antibiotics have strong recommendation power [4].

As previously described, tretinoin is susceptible to degradation upon exposure to various factors. Notably, when combined with benzoyl peroxide in the liquid phase, it undergoes oxidation through free radical–mediated degradation. Because the combination of tretinoin with benzoyl peroxide has demonstrated substantial clinical benefit, a corresponding technological innovation has emerged. It was described in a work by Hakak et al. [36] where the process of microencapsulation of tretinoin and benzoyl peroxide protects tretinoin against the oxidizing potential of benzoyl peroxide and enables the creation of a stable formulation.

Although this combination provides superior efficacy in acne treatment, concerns regarding an increased risk of local adverse effects may arise. In a study by Poteate et al. [37], the side effect profile of encapsulated benzoyl peroxide 3%/tretinoin 0.1% was compared with that of encapsulated benzoyl peroxide 5% alone. Typical local adverse effects associated with topical therapy were reported in a relatively small proportion of patients and were not clearly more frequent in the combination group. Moreover, owing to the greater clinical efficacy of the combined formulation, treatment adherence appeared to be higher. This combination, therefore, represents a major technological achievement, reaching commercialization many years after the initial introduction of tretinoin.

Another beneficial therapeutic approach is the combination of a retinoid with a topical antibiotic. As stated in current recommendations, topical antibiotics should not be used as monotherapy in the treatment of acne vulgaris [4]. Consequently, recent studies have predominantly focused on combinations of tretinoin with clindamycin.

In the study by Dogra et al. [38], a combination of tretinoin with clindamycin using microspheres technology was compared to these agents as monotherapy. A superior therapeutic effect of the combination treatment was demonstrated, highlighting the advantages of microsphere technology, which allows enhanced intralesional absorption of tretinoin while reducing its irritation potential.

Considering the various combinations of retinoids with other agents, the question arises as to which regimen offers the greatest therapeutic benefit. Comparative studies of different combination therapies are also available. For example, Aschoff et al. [39] compared a tretinoin/clindamycin regimen with adapalene/benzoyl peroxide and found that both options demonstrated similar efficacy in mild to moderate papulopustular acne. However, treatment with the tretinoin/clindamycin combination was associated with fewer adverse effects, which may translate into better treatment adherence. Nevertheless, further studies are needed, and the choice of an appropriate combination should be guided by individual patient characteristics and prior treatment history.

Remaining in the topic of combining tretinoin with other substances, an interesting study was conducted by Bertolani et al. [40], in which the effects of combining tretinoin 0.02% and clindamycin 0.8% with 4% glycolic acid in a gel formulation were examined in various application modalities. Standard therapy with such a gel was described earlier in a study by Milani et al. [41] showing promising results, but 16% dropout rate due to poor local tolerability was noted. Because of that, an idea of a modified application emerged, which was described by Bertolani et al. [40]; one group of patients used this formulation in short contact therapy (SCT) mode (complete removal of product after 1 h) and another in standard application therapy (SAT) mode. All patients had mild to moderate acne. Obtaining promising results in the SCT group, the authors suggest that this mode of application may be advantageous in reducing the risk of irritation while maintaining at least comparable clinical efficacy to standard application.

Innovative approaches have also been proposed that combine procedural interventions, such as fractional carbon dioxide (CO_2_) laser therapy, with tretinoin treatment. In their exploratory study, Pestoni Pórven et al. [42] have demonstrated the high effectiveness of such a therapeutic approach in 2 patients, one with nodulocystic and the second with microcystic acne. The underlying mechanism of this approach involves the creation of numerous microchannels in the skin by the laser; these channels initiate a skin-remodeling cascade and simultaneously facilitate the penetration and activity of tretinoin. Although the study demonstrated favorable results, it should be noted that there are very few studies examining the combination of tretinoin or other topical retinoids with medical technologies. This area, therefore, requires further investigation.

## 4. Third Generation of Topical Retinoids—Adapalene and Tazarotene

Adapalene and tazarotene are topical representatives of this group. They are synthetic retinoid analogues and already have a well-established position in acne vulgaris and acne scarring treatment. Their mechanism of action is more selective than that of tretinoin. Adapalene and tazarotene both interfere mainly with beta and gamma retinoic acid receptors. Tazarotene, being a pro-form that is being metabolized to tazarotenic acid, affects these receptors with greater affinity, which results in high potency, but also a greater risk of developing retinoid irritant dermatitis [43,44]. Since its introduction in 1996, adapalene has undergone substantial formulation development, progressing from solutions and gels to creams and lotions, with available concentrations ranging from 0.1% to 0.3% [1].

Currently, gel and cream formulations are the most commonly used, with cream-based vehicles generally considered more suitable for patients prone to irritation and those with sensitive skin. Such a preference has also been confirmed in a study by Dosik et al. [45], where comparison has been made not only between gel and cream, but also between tazarotene cream at concentrations of 0.05% and 0.1%. The irritancy assessment was based on a cumulative irritation assessment performed under occlusive conditions, in which test products were applied to the upper back for approximately 24 h four times per week and for 72 h once weekly over a 3-week period. Skin reactions (including erythema scores and other local reactions) at the application sites were assessed 5 to 30 min after dressing removal. The study clearly demonstrated a graded irritancy potential, as follows: adapalene 0.1% cream < adapalene 0.1% gel < tazarotene 0.05% cream < tazarotene 0.1% cream. Similar comparisons between adapalene gel and cream and various tretinoin formulations were performed in a study by Brand et al. [46]. In that study, adapalene 0.1% cream demonstrated the lowest irritancy potential compared with the gel formulation, while tretinoin formulations showed progressively higher irritancy indices with increasing concentrations. Interestingly, marked differences in pharmacokinetics can be observed even within the same formulation type. In a study by Matharoo et al. [47], which evaluated three different generic 0.1% adapalene gels, one formulation demonstrated a superior release rate and higher dermal delivery compared with the others. These findings highlight how even subtle differences in formulation composition can significantly influence pharmacokinetics.

Because minimizing the risk of adverse effects from topical formulations remains a priority, thereby improving treatment adherence, novel formulations that combine improved tolerability with enhanced efficacy continue to be investigated. One such approach involves NLCs, a technology also explored for tretinoin delivery. NLCs are lipid-based transporters composed of lipids, surfactants, and co-surfactants, with particle sizes typically ranging from 50 to 500 nm, enabling improved penetration through the stratum corneum in a controlled manner [48]. In a study by Ahmad et al. [49], the prepared formulation was tested in a cohort of 15 patients over 12 weeks, demonstrating clinical improvement with good safety and tolerability in the majority of participants.

Another novel technology described in recent years is microemulsions. Shao et al. [50] reported the development of a stable adapalene-loaded formulation with good physicochemical properties, good dermal retention, and adequate permeability, suggesting this formulation as a potential alternative to conventionally used preparations.

Using a medium that enables the gradual and controlled release of an active substance remains an appealing option. One such innovative platform is microsponge technology. Microsponges are porous structures capable of entrapping active compounds and are engineered to allow controlled release of active substances. In a study by Yehia et al. [51], adapalene-loaded microsponges were incorporated into a hydrogel formulation. Owing to the promising results observed in a small cohort of 10 patients with mild to moderate acne, the authors suggested that future commercialization of this approach may be feasible.

Another interesting class of nanocarriers is niosomes, which are vesicular systems composed of non-ionic surfactants and cholesterol that are capable of encapsulating a wide variety of active substances while maintaining a favorable safety profile [52,53]. In a study by Tabatabaie-Mehr et al. [54], niosomes have been used as carriers for adapalene incorporated into a hydrogel formulation. This approach resulted in a formulation with good stability, favorable skin penetration potential, and controlled drug release. Importantly, the authors reported no signs of irritation in rabbit skin studies using the tested hydrogel.

The chemical structure of adapalene is much more resistant to the oxidative effects of benzoyl peroxide than that of tretinoin. For this reason, such a synergistic combination could be introduced much earlier, without the need for complex technological processes such as those required for tretinoin. As it was mentioned before, such a combination has beneficial properties, which have been confirmed in numerous studies over the years in various groups of patients with mild to severe acne [55,56,57,58]. Additionally, focusing on the most challenging group of patients—the ones with severe acne, Del Rosso et al. [59] observed a positive effect of adapalene and benzoyl peroxide combined with oral doxycycline as an alternative for patients who are candidates for isotretinoin treatment.

Although the superior synergistic effect of combining a topical retinoid with a topical antibiotic has been well established, it should be noted that, according to recent American Academy of Dermatology guidelines on multimodal acne treatment, the addition of benzoyl peroxide to such combinations is recommended [4]. To date, the only commercially available triple combination therapy consists of adapalene 0.15%, benzoyl peroxide 3.1%, and clindamycin phosphate 1.2%. This formulation provides high therapeutic potency while reducing the risk of antibiotic resistance [60]. It should be kept in mind that formulations combining a retinoid with a topical antibiotic are generally recommended for no longer than 12 weeks of therapy because of the increased risk of antibiotic resistance. An interesting study by Ghannoum et al. [61] evaluated a 24-week therapy with a triple combination of adapalene/benzoyl peroxide/clindamycin, reporting good effectiveness, tolerability, and no evidence of bacterial resistance development. Evidently, adapalene stands out with its stability, resistance to benzoyl peroxide, and high tolerability, giving it an established position in acne treatment.

Introduced in 1997, tazarotene has been available in gel, cream, foam, and lotion formulations, predominantly at a concentration of 0.1%, with the exception of a novel 0.045% lotion introduced in 2019 [1]. Given the higher irritancy potential of tazarotene compared with adapalene [45], polymeric emulsion technology has been developed to maximize its therapeutic efficacy while reducing the risk of treatment discontinuation due to adverse effects. Such an emulsion of mesh structure ensures controlled active substance release. The irritation potential of this polymeric emulsion was evaluated by Draelos et al. [62], who compared tazarotene 0.045% lotion with adapalene 0.3% gel and trifarotene 0.005% cream. Irritation patch tests were performed, and the tazarotene-loaded emulsion appeared to be the least irritant from the examined formulations. Pediatric patients are the group in whom a low irritation potential is of greatest importance, as it enables continued effective treatment without compromising quality of life. A study by Eichenfield et al. [63] focused on patients aged 10–13 and 14–17 years with moderate to severe acne. Over 12 weeks of once-daily treatment with tazarotene 0.045% lotion, the therapy was found to be effective and well-tolerated in these patient groups.

Another tazarotene formulation at a different concentration, namely 0.05% gel, has also been evaluated. In a study by Deshmukh et al. [64], this formulation was compared with adapalene 0.1% gel, revealing a faster onset of action and greater apparent efficacy for tazarotene, albeit with a higher incidence of adverse effects.

Microemulsions have entered the dermatology world as stable formulations, with good active substance absorption potential and stability. A study by Badawi et al. [65] evaluated a tazarotene-loaded microemulsion using both in vitro and in vivo models. The authors described the development and characterization of a stable microemulsion with improved skin deposition and, consequently, enhanced activity. However, the experiments were conducted in an animal model, and additional data from human clinical trials are needed.

A recent study by Kshirsagar et al. [66] focused on developing another nanotechnological approach to tazarotene delivery using tazarotene-loaded poly(lactic-co-glycolic acid) (PLGA) nanoparticles. During nanoparticle preparation, tazarotene is entrapped within the PLGA matrix, enabling efficient follicular delivery while maintaining prolonged, controlled contact with the skin, thereby reducing the risk of irritation. The selection of PLGA was justified by its favorable biocompatibility and biodegradability. The diversity of tazarotene formulation strategies described in recent studies clearly demonstrates the substantial potential for further development in this field.

Although no commercially available formulation combining tazarotene with an antibiotic currently exists, results of studies evaluating the efficacy of such combinations have been reported. Tanghetti et al. [67] confirmed the superior efficacy of combining benzoyl peroxide/clindamycin gel with tazarotene gel compared with tazarotene monotherapy in patients with moderate to severe acne. According to the authors, no patients discontinued treatment because of adverse effects or lack of efficacy. In contrast, a study by Dhawan et al. [68] examined tazarotene combined with clindamycin and benzoyl peroxide at two different concentrations (2.5% and 5%). Both regimens were effective; however, the combination containing 5% benzoyl peroxide was associated with greater transient irritation at week 4, while by week 8, both treatments demonstrated comparable tolerability.

The advantage of combination therapy is well established and supported by both clinical guidelines and comparative studies evaluating different retinoid-based combinations to identify the most effective options. In another study, Tanghetti et al. [69] compared the effectiveness of tazarotene/clindamycin with that of tretinoin/clindamycin and demonstrated the superiority of the tazarotene/clindamycin combination. Similarly, Maiti et al. [70] compared tazarotene/clindamycin with the well-established adapalene/clindamycin regimen, again showing greater efficacy for tazarotene/clindamycin. The observed superiority of tazarotene may be explained by its strong anti-inflammatory and keratinization-regulating effects, attributable to its high affinity for RAR-β and RAR-γ receptors, which may further enhance comedolytic activity and improve antibiotic penetration.

## 5. Fourth Generation of Topical Retinoids-Trifarotene

Trifarotene is currently the only representative of this class and was officially approved in 2019. This synthetic molecule has a strong affinity for RAR-γ, the most abundant RAR subtype in the skin. Its selective activity, characterized by high cutaneous potency and minimal risk of systemic exposure, allows for safer use over larger surface areas, including in the treatment of truncal acne [71,72].

One of the first reports on trifarotene published in 2018 by Thoreau et al. [73] describes the development of this novel selective molecule based on structure–activity and structure–chemical correlations. Subsequently, in 2019, Tan et al. [74] reported results from one of the first large-scale clinical trials evaluating trifarotene 50 µg/g in the treatment of moderate facial and truncal acne. In this study, more than a thousand patients were randomized to the trifarotene and placebo groups, with the trifarotene group showing favorable results.

Because acne therapy often requires prolonged retinoid use to maintain therapeutic outcomes, studies evaluating the long-term safety of such treatments have also been conducted. Blume-Peytavi et al. [75] confirmed the safety and satisfactory tolerability of trifarotene during yearlong use. In a large group of patients, a high completion rate was observed. As expected, better tolerability was observed on the trunk than on the face, which is understandable given the greater epidermal thickness in truncal skin. Importantly, typical local adverse effects occurred mainly at the beginning of therapy and tended to subside over time, consistent with the characteristic retinization process [75,76]. A later study by Dreno et al. [77] investigated processes on the molecular level and changes in gene expression related to trifarotene. Biopsied lesions treated with trifarotene were also evaluated, revealing 67 genes that were modulated by trifarotene but not affected in spontaneously resolving lesions. This innovative study highlights the complex and multifactorial molecular changes involved in the therapeutic response.

As trifarotene has become an established agent for the treatment of both facial and truncal acne, additional studies have emerged evaluating its use in more diverse patient populations.

One of them comprises patients with higher phototypes, who are more susceptible to developing post-inflammatory hyperpigmentation. Del Rosso et al. [78] evaluated the effectiveness of trifarotene with and without doxycycline in patients with phototypes III and higher, reporting favorable outcomes and satisfactory tolerability. However, as the study included only five patients, further research involving larger cohorts is required.

Emerging strategies involving trifarotene focus on its integration into multimodal treatment approaches targeting both active acne and its sequelae. Due to its high selectivity for retinoic acid receptor gamma (RAR-γ), trifarotene exerts targeted effects on epidermal differentiation and inflammation, making it a promising option for long-term management of acne-induced post-inflammatory hyperpigmentation [79]. Beyond monotherapy, trifarotene has been proposed as an adjunct in acne scar treatment, where its remodeling effects complement procedural interventions such as laser therapy or microneedling [80]. Sequential combination strategies have also been explored, including the use of topical trifarotene followed by injectable non-animal stabilized hyaluronic acid (NASHA) gel, demonstrating significant clinical improvement in atrophic acne scars, likely due to synergistic stimulation of collagen remodeling [81]. These findings suggest that trifarotene may play an important role not only in acne control but also in comprehensive, long-term management of acne-related skin changes. However, we did not find studies that evaluate the combination of trifarotene with benzoyl peroxide or clindamycin.

Schematic illustrations for retinoid monotherapy and combination strategies by retinoid generation are presented in Table 2.

## 6. Acne Scar Treatment with Topical Retinoids

The formation of acne lesions, beginning with microcomedones and progressing to more advanced lesions, involves varying degrees of inflammation. This inflammatory process can ultimately lead to acne scarring, which may be atrophic (the most common consequence of acne lesions) or hypertrophic. The hallmark of scar formation is dysbalanced collagen production and chaotic tissue architecture [82,83]. Because topical retinoids modulate fibroblast activity and reduce inflammation, they can promote tissue remodeling [84]. All of the retinoids discussed above have been investigated for their effects on acne scarring. In addition to acne severity, patient-related factors, such as individual susceptibility to scar formation, should also be taken into account [85].

Considering all the sequelae of acne and the risk of acne scar formation, the potential of tretinoin in the reduction of such lesions has been studied through the years. Early studies described the beneficial effects of tretinoin on hypertrophic scars [86]. Subsequently, as its mechanism of action became better understood, particularly its strong stimulation of collagen I and III synthesis and the improvement in the organization of newly formed collagen bundles, tretinoin was also shown to help improve the appearance of atrophic acne scarring as well as post-inflammatory hyperpigmentation (PIH) [87,88].

Only a few publications have reported the favorable effects of combining tretinoin with iontophoresis in the treatment of atrophic acne scarring. Iontophoresis is a technique used to enhance transdermal drug delivery by applying low-intensity electrical currents [89].

The first study by Schmidt et al. compared iontophoresis with tretinoin and estriol, reporting clinical improvement in both treatment groups, although several patients in the tretinoin group developed symptoms of skin irritation [90]. Subsequently, another study by Schmidt et al. [91], focusing exclusively on tretinoin, demonstrated clinical improvement in young patients with atrophic acne scarring. In addition, Knor [92] reported flattening of atrophic scars in 79% of treated patients following a series of iontophoresis sessions using 0.05% tretinoin gel, supplemented with three to four mild trichloroacetic acid (TCA) peels during the treatment protocol. No further large-scale studies on this approach have been identified, possibly reflecting the emergence of newer technologies such as laser therapy and microneedling, as well as a decline in the use of iontophoresis in subsequent years.

Tretinoin was also used in a complex scar treatment protocol as a conditioning agent in the study by Garg and Baveja [93]. Their study aimed to assess a complex protocol for acne scar treatment, which involved the use of tretinoin 0.05% cream for 2 weeks prior to procedures such as scar subcision, microneedling in combination with tretinoin 0.05% cream one day after subcision and leaving the tretinoin cream for 30 min after the procedure. Finally, a 15% TCA peel was applied two weeks after the microneedling procedure.

The protocol included the use of tretinoin cream as part of a night-time regimen, with appropriate treatment-free intervals before repeating specific procedures. The study design assumed six alternating sessions of microneedling and TCA peeling. This protocol was particularly targeted at patients with severe scarring, and notably, tretinoin played a crucial role within the comprehensive treatment plan. The cited study reported high levels of patient satisfaction and demonstrated beneficial effects not only in individuals with grade 2 scars, but also in those with grade 3 and 4 scars. Moreover, this treatment approach was considered suitable for patients with higher skin phototypes.

The third generation of topical retinoids, such as adapalene and tazarotene, has also been investigated regarding their effect on acne scarring. An interesting study by Loss et al. [94] focused on the effects of long-term maintenance therapy with adapalene, which lasted 24 weeks. Adapalene at a concentration of 0.3% was tested in a group of 20 patients, showing improvement in skin texture ranging from 1 to 2 grades from baseline in 55.6% of subjects, resulting in an increase in the patients’ quality of life.

Another long-term 24-week study by Tanizaki et al. [95] focused on the comparison of adapalene 0.1%/benzoyl peroxide 2.5% gel to benzoyl peroxide 2.5% gel alone in maintenance therapy. Three-dimensional image analysis was used to evaluate skin lesions. Interestingly, the study demonstrated a similar reduction in the progression of acne scarring in both the adapalene/benzoyl peroxide group and the benzoyl peroxide monotherapy group, compared with the placebo group, in which scarring worsened. This suggests that both the retinoid–benzoyl peroxide combination and benzoyl peroxide alone exert anti-inflammatory effects that may help prevent further scar development.

A study summarizing the effects of tazarotene on acne scarring was conducted by Afra et al. [96]. In this trial, 36 patients were enrolled and treated for three months, with one half of the face receiving daily topical tazarotene 0.1% and the other half undergoing dermaroller treatment, consisting of four procedures over the same period. The authors reported a significant reduction in scarring in both treatment arms, suggesting that topical tazarotene may represent an effective alternative to microneedling for the management of acne scars. On the other hand, a case report published by Miranti [97] confirmed that the 12-month treatment with tazarotene 0.045% lotion improved scar appearance, demonstrated good maintenance of anti-acne treatment outcomes, and showed favorable tolerability.

Although trifarotene remains a relatively new drug, its effects on acne scarring have already been investigated. In a study by Schleicher et al. [98], more than 100 patients were enrolled and randomized to trifarotene or placebo. Over the 24-week study period, a significant reduction in acne scarring was observed in the trifarotene group compared to placebo. In another case series, Belmontesi [81] investigated a combined approach using trifarotene and non-animal stabilized hyaluronic acid (NASHA) gel. SCT with once-daily trifarotene for three months was followed by treatment with NASHA gel at a concentration of 20 mg/mL, used as a skin booster, administered in three to ten sessions. A significant clinical improvement was observed, which the authors attributed to a synergistic effect of the topical retinoid and NASHA gel on skin remodeling.

## 7. Nanotechnology

Advanced nanotechnology-based delivery systems enhance the efficacy of topical retinoids through multiple complementary mechanistic pathways involving improved penetration, targeted follicular delivery, and controlled drug release. Lipid-based carriers such as NLCs and solid lipid nanoparticles (SLNs) possess a core–shell architecture in which the drug is incorporated within a lipid matrix stabilized by surfactants, enabling protection from degradation and sustained release kinetics [99]. The imperfect crystalline structure of NLCs, resulting from the combination of solid and liquid lipids, increases drug loading capacity and prevents drug expulsion, while simultaneously enhancing diffusion through the stratum corneum lipid matrix [34]. Vesicular systems such as niosomes further facilitate penetration via their bilayer structure, allowing incorporation of lipophilic retinoids within the membrane and hydrophilic components in the aqueous core, thereby improving partitioning into skin layers and enabling controlled release [54]. Importantly, these carriers enhance retention within the pilosebaceous unit, a key target site in acne pathogenesis. Polymeric nanoparticles, such as PLGA-based systems, provide an additional mechanistic advantage by enabling diffusion-controlled drug release (e.g., Higuchi kinetics) and prolonged residence time, while maintaining drug bioconversion to active metabolites within the skin [66]. All those features, such as nanoscale size, high surface area, and optimized physicochemical interactions with the skin barrier, improve retinoid bioavailability, enhance therapeutic targeting, and reduce irritation through controlled exposure.

## 8. Benefits of Combination Strategies for Treatment of Acne and Acne Scars

Combination therapy constitutes a cornerstone of contemporary acne management, as it enables simultaneous targeting of multiple pathogenic mechanisms, including follicular hyperkeratinization, inflammation, and microbial proliferation [93,100]. The combination of topical retinoids with benzoyl peroxide remains a first-line approach, offering comedolytic and anti-inflammatory effects while reducing the risk of *C. acnes* resistance [61]. Similarly, retinoid–antibiotic combinations, such as tretinoin or adapalene with clindamycin, enhance antimicrobial and anti-inflammatory efficacy, although the addition of benzoyl peroxide is recommended to mitigate antibiotic resistance. Recently introduced triple-combination formulations (e.g., adapalene/benzoyl peroxide/clindamycin) further simplify treatment regimens while maintaining high efficacy and adherence [36,37,39]. Advanced formulation strategies, including nanostructured lipid carriers and polymeric emulsions, improve drug stability, penetration, and tolerability, thereby supporting long-term use [48,49]. Beyond pharmacological approaches, combining topical retinoids with procedural interventions, such as microneedling, chemical peels, or fractional laser therapy, has demonstrated synergistic benefits, particularly in patients with acne scarring, by enhancing collagen remodeling and drug delivery [42,88,93]. Additionally, integration with systemic therapies, including oral antibiotics or hormonal agents, may be indicated in moderate to severe cases, followed by topical maintenance [97,101]. Overall, combination therapy not only improves clinical outcomes but also optimizes tolerability, reduces resistance risk, and enhances patient adherence, making it a fundamental strategy in acne treatment.

## 9. Practical Implications

Topical retinoids should be considered a foundational therapy across all stages of acne, with selection tailored to patient characteristics, including skin sensitivity, age, and disease severity. Combination therapy, particularly with benzoyl peroxide and, when indicated, topical or systemic agents, is supported by current guidelines to improve efficacy and limit antibiotic resistance. Newer formulations (e.g., polymeric emulsions, nanocarriers) offer improved tolerability and may enhance adherence, particularly in patients prone to irritation. Combination therapy, especially with benzoyl peroxide and/or antibiotics, should be preferred over monotherapy to increase efficacy and reduce the risk of bacterial resistance. In patients with acne scarring or higher risk of sequelae, early introduction of retinoids and their integration with procedural treatments (e.g., microneedling, chemical peels, laser) may improve long-term outcomes. Advanced strategies, including controlled-release systems and sequential therapies, enable optimization of efficacy while minimizing adverse effects. Newer delivery systems and multimodal scar-directed approaches are promising, but at present they should be viewed as adjunctive or emerging strategies rather than core guideline-mandated interventions. Overall, individualized, multimodal approaches that combine pharmacological, technological, and procedural modalities represent the most effective strategy in modern acne and acne scar management.

## 10. Conclusions

Topical retinoids represent an effective therapeutic option in the management of acne vulgaris and acne scarring, ranging from monotherapy in cases of mild lesions to incorporation into more complex, multimodal treatment protocols for patients with more severe lesions. The available evidence suggests beneficial effects across different retinoid molecules and delivery technologies, including improvements in scar appearance, skin remodeling, and maintenance of acne control. Evidence supporting novel formulations varies, with some evaluated in large studies and others only in small heterogeneous populations or at the preclinical stage. While topical retinoids are supported by numerous publications over the years, novel formulations still require well-designed, large-scale comparative studies to better define the relative efficacy, tolerability, and optimal clinical positioning in the treatment of acne and acne scarring.

## Figures and Tables

**Table 1:** Retinoids used topically–mechanism of action and irritation potential [3,7,8,9,10,11,12].

Generation	Name	Structure	Mechanism of Action	Irritation Potential
1st	Tretinoin	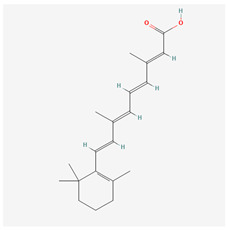	Activation of RARα, RARβ, RARγ Downregulation of MMPs Suppression of tyrosinase AP-1 inhibition NF-κB inhibition Stimulation of TGF-β signaling	High
3rd	Adapalene	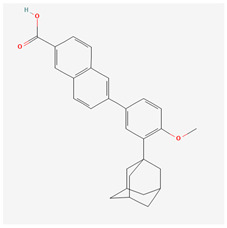	Activation of RARβ and RARγ Downregulation of MMPs Suppression of tyrosinase AP-1 inhibition NF-κB inhibition Stimulation of TGF-β signaling	Low–moderate
	Tazarotene	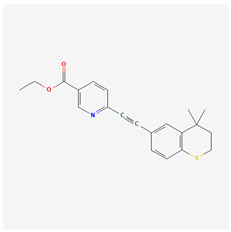	Activation of RARβ and RARγ Downregulation of MMPs Suppression of tyrosinase AP-1 inhibition NF-κB inhibition Stimulation of TGF-β signaling	High
4th	Trifarotene	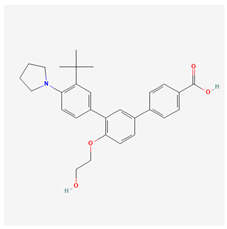	Highly selective RARγ activation Downregulation of MMPs Suppression of tyrosinase AP-1 inhibition NF-κB inhibition Stimulation of TGF-β signaling	Moderate

Abbreviations: MMPs—matrix metalloproteinases, AP-1—activator protein 1, NF-κB—nuclear factor kappa-light-chain-enhancer of activated B cells, TGF-β—transforming growth factor beta.

**Table 2:** Schematic illustrations for retinoid monotherapy and combination strategies in acne vulgaris.

Generation	Name	Possible Combinations	Technologies
1st	tretinoin	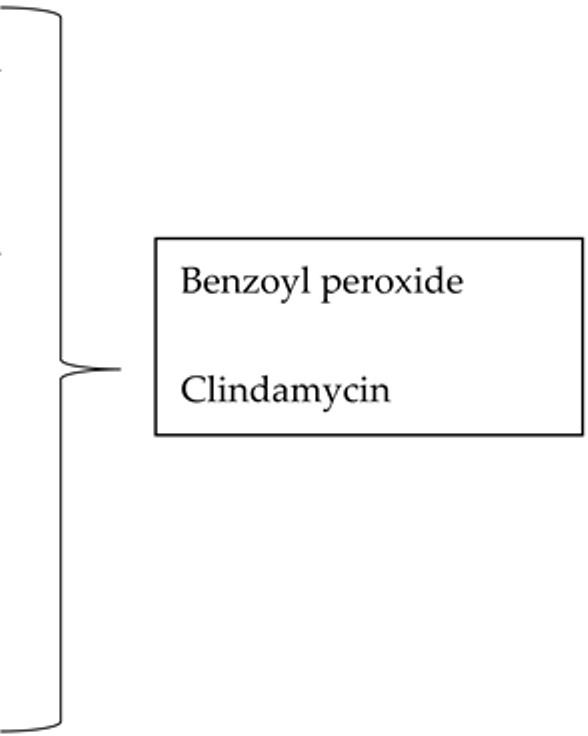	Encapsulation technology for combination with benzoyl peroxide. Microsphere technology for combination with clindamycin.
3rd	adapalene		The only retinoid currently commercially available in combination with benzoyl peroxide and clindamycin in a single formulation.
	tazarotene		No commercially available combinations of benzoyl peroxide/clindamycin with tazarotene. Advantageous effects are described in the studies.
4th	trifarotene		No data so far regarding combination therapy with benzoyl peroxide or clindamycin.

## Data Availability

No new data were created or analyzed in this study. Data sharing is not applicable to this article.

## Abbreviations

The following abbreviations are used in this manuscript:

AP-1	Activator Protein 1
ATRA	All-trans retinoic acid
CO_2_	Carbon dioxide
MMP	Matrix metalloproteinase
NASHA	Non-animal stabilized hyaluronic acid
NF-kB	Nuclear factor kappa-light-chain-enhancer of activated B cells
NLC	Nano lipid carriers
PIH	Post-inflammatory hyperpigmentation
PLGA	Polylactic co-glycolic acid
RAR	Retinoic acid receptor
RXR	Retinoid X receptor
SAT	Standard application therapy
SCT	Short contact therapy
TCA	Trichloroacetic acid
TGF-b	Transforming growth factor beta
